# Editorial: Infectious Diseases Affecting Reproduction and the Neonatal Period in Cattle

**DOI:** 10.3389/fvets.2021.679007

**Published:** 2021-04-22

**Authors:** Dadín P. Moore, Germán J. Cantón, Enrique L. Louge Uriarte

**Affiliations:** ^1^Animal Production Department, Institute of Innovation for Agricultural Production and Sustainable Development (IIPADS), Balcarce, Argentina; ^2^Faculty of Agricultural Sciences, National University of Mar del Plata, Mar del Plata, Argentina; ^3^Animal Production Department, National Institute of Agricultural Technology (INTA), Balcarce, Argentina

**Keywords:** cattle, bovine abortion, neonatal mortality, diagnosis, production system

Even with the global scenario after the SARS CoV-2 pandemic, human population keeps growing, and therefore food safety and quality demand is increasing. So, it is required to improve the efficiency in most livestock production systems including the cattle industry. Because the efficiency of cattle industry is far away from optimum (1–3), the intensification of the production systems emerges as a challenge. Currently, over 1 billion heads are raised in our planet. Countries like Argentina, Australia, Brazil, China, and United States extensively raise their cattle on pastures, which represents over 50% of the productive cattle stock worldwide. The main objective of cow-calf systems is to produce the largest quantity of calves per bred cow. Nevertheless, top beef producing countries in some cases achieve only above 50% of weaning rate. Common causes of this low weaning rate usually occur during the breeding season. In this period, cows are usually under suboptimal body condition, exposed to environmental stress and/or infectious diseases, and therefore low pregnancy rates are recorded. The diagnosis of the cause of this early reproductive failure is challenging, unless they are related with infectious diseases. Many research articles reports abortion and perinatal mortality varying from 5 to 12% and 2 to 5%, respectively (4–8) representing a huge loss of calves.

During the period from pregnancy diagnosis to calf delivery, the efficiency of detecting the aetiological agents or diseases is still below 50% even though several studies and experimental models on this topic have been developed. Moreover, even when control, management, vaccination, and drug treatments are available, many risk factors still have a negative impact during the pregnancy and perinatal periods (3, 7, 9). Low conception rates, subfertility or stillbirths in cattle can be associated with different causes but the diagnosis of them is not always easy, either because the appropriate and specific sample was not sent or simple “an improper labeling.” Animal welfare must also be taken into account in every livestock production system. Reproductive efficiency is a direct indicator of the health and welfare situation of your animals. Therefore, low reproductive rates (prolonged anestrus, low conception rates, high reproductive losses, and high percentage of assisted deliveries and/or dystocia) may indicate animal welfare problems. Indeed, differential diagnosis is critical and essential to identify the causes of reproductive losses and perinatal mortality in cattle.

Anamnesis is the first step in diagnosis. It should include the bull, the dam and its progeny. Whether the losses are sporadic or seem to be an outbreak (more than 10% of the herd affected during 45–60 days) may not only be associated with the occurrence of endemic or epidemic diseases but also the agent causing abortions or perinatal mortality. The existence of Animal Health Programs and the vaccination schedule must be requested. Secondly, serology can be assessed for studying affected animals and controls, then association among event and serological results can be statistically tested (9). Over 40 years, the Animal Health Group at INTA, Balcarce, Argentina, has been successful in detecting reproductive losses in the herds by following a sentinel group of females between the time of the pregnancy test and delivery. Both transrectal pregnancy test and blood sampling are performed monthly, and cervical-vaginal swabs are obtained from aborted cows/heifers. Third, the differential diagnosis must be based on running methodic diagnostic tests on different samples at the laboratory (6, 10). Here, a thorough fetal necropsy and carefully sampling is essential (9). Several laboratories performing bacteriology, virology, toxicology, biochemistry, and histopathology must be involved to perform a proper differential diagnosis. Finally, everyone including farmers, veterinarians, Lab's technicians, and researchers must work as a team to arrive at a diagnosis as fast as possible.

Bovine abortion and perinatal mortality have multifactorial origin but they can be classified in: genetic, environmental and infectious (including parasites). Indeed, differential diagnosis gets relevance because the diversity of causes and risk factors involved (9). Genetic causes include chromosome or gene abnormalities but most of them are beyond routine diagnosis. The causes of environmental origin are poorly reported and probably underdiagnosed. They may include traumatic abortions, toxic, hormonal, nutritional (mineral and vitamin deficiencies), unusual high temperatures specially during the breeding season, and mechanical factors (uterine torsion, umbilical cord compression). Infectious agents represent 50% of the identified causes either for abortion or perinatal mortality. Brucellosis (4), campylobacteriosis (4), and leptospirosis (5) are the main bacterial causes of bovine abortion. Moreover, some of these bacterial reproductive diseases are zoonotic, therefore special caution and effort should be taken in order to prevent them. Viral agents are bovine viral diarrhea virus, bovine herpesvirus-1 (BHV-1) and more recently described, bovine herpesvirus-4, which associated with bacteria may cause infertility (2, 11). Among protozoal agents, *Tritrichomonas fetus* and *Neospora caninum* are responsible of embryo deaths and abortions, respectively (4, 12). Fungal infections associated with abortions are usually sporadic but no less important (6). Noteworthy, co-infections may be more frequent and relevant than previously though. Several studies report the occurrence of co-infections: many miscellaneous bacteria (4), *Leptospira* spp. and *N. caninum* (5), BHV-1 and *N. caninum* (13). This may be even more challenging and careful recommendations for controlling and preventing several diseases must be taken.

Differential diagnosis is essential but similar effort must be done to follow the outcome according to given recommendations. Although many advances have been achieved including modern molecular techniques, an effort to teach and transfer this available knowledge to students, young researchers and veterinary practitioners must be performed to prevent and control diseases affecting reproduction and the perinatal period in cattle.

## Author Contributions

DM, GC, and EL conception of the work, critical revision of the work for important intellectual content, and final approval of the version to be published. All authors contributed to the article and approved the submitted version.

## Conflict of Interest

The authors declare that the research was conducted in the absence of any commercial or financial relationships that could be construed as a potential conflict of interest.

## References

[1] Reese T Franco GA Poole RK Hood R Fernadez Montero L Oliveira Filho RV. Pregnancy loss in beef cattle: a meta-analysis. Anim Reprod Sci. (2020) 212:106251. 10.1016/j.anireprosci.2019.106251 31864492

[2] Wathes DC Oguejiofor CF Thomas C Cheng Z. Importance of viral disease in dairy cow fertility. Engineering. (2020) 6:26–33. 10.1016/j.eng.2019.07.020 32288965 PMC7104734

[3] Cuttance E Laven R. Estimation of perinatal mortality in dairy calves: a review. Vet J. (2019) 252:105356. 10.1016/j.tvjl.2019.105356 31554595

[4] Campero CM Moore DP Odeón AC Cipolla AL Odriozola E. Aetiology of bovine abortion in Argentina. Vet Res Commun. (2003) 27:359–69. 10.1023/A:1024754003432 14509450

[5] Corbellini LG Pescador CA Frantz F Wunder E Steffen D Smith DR. Diagnostic survey of bovine abortion with special reference to *Neospora caninum* infection: importance, repeated abortion and concurrent infection in aborted fetuses in Southern Brazil. Vet J. (2006) 172:114–20. 10.1016/j.tvjl.2005.03.006 16772136

[6] Clothier K Anderson M. Evaluation of bovine abortion cases and tissue suitability for identification of infectious agents in California diagnostic laboratory cases from 2007 to 2012. Theriogenology. (2016) 85:933–8. 10.1016/j.theriogenology.2015.11.001 26679514

[7] Morrell EL Moore DP Odeón AC Poso MA Odriozola E Cantón G. Retrospective study of bovine perinatal mortality: cases reported from INTA Balcarce, Argentina. Rev Argent Microbiol. (2008) 40:151–7. 19024501

[8] Norquay R Orr J Norquay B Ellis KA Mee JF Reeves A. Perinatal mortality in 23 beef herds in Orkney: incidence, risk factors and aetiology. Vet Rec. (2020) 187:28. 10.1136/vr.105536 33638491 PMC7456677

[9] Mee JF. Investigation of bovine abortion and stillbirth/perinatal mortality – similar diagnostic challenges, different approaches. Irish Vet J. (2020) 73:20. 10.1186/s13620-020-00172-0 32944225 PMC7487920

[10] Tramuta C Lacerenza D Zoppi S Goria M Dondo A Ferroglio E. Development of a set of multiplex standard polymerase chain reaction assays for the identification of infectious agents from aborted bovine clinical samples. J Vet Diagn Invest. (2011) 23:657–64. 10.1177/1040638711407880 21908306

[11] Chen X Wang X Qi Y Wen X Li C Liu X. Meta-analysis of prevalence of bovine herpes virus 1 in cattle in Mainland China. Acta Trop. (2018) 187:37–43. 10.1016/j.actatropica.2018.07.024 30055174

[12] McAllister MM. Diagnosis and control of Bovine Neosporosis. Vet Clin North Am Food Anim Pract. (2016) 32:443–63. 10.1016/j.cvfa.2016.01.012 27161392

[13] Rinaldi L Pacelli F Iovane G Pagnini U Veneziano V Fusco G. Survey of *Neospora caninum* and bovine herpes virus 1 coinfection in cattle. Parasitol Res. (2007) 100:359–64. 10.1007/s00436-006-0335-4 17053931

